# Circular RNAs Hsa_circ_0002715 and Hsa_circ_0035197 in Peripheral Blood Are Novel Potential Biomarkers for New-Onset Rheumatoid Arthritis

**DOI:** 10.1155/2019/2073139

**Published:** 2019-10-29

**Authors:** Qing Luo, Jun Liu, Biqi Fu, Lu Zhang, Yang Guo, Zikun Huang, Junming Li

**Affiliations:** ^1^Department of Clinical Laboratory, The First Affiliated Hospital of Nanchang University, Nanchang, Jiangxi 330006, China; ^2^Department of Medical College, Nanchang University, Nanchang, Jiangxi 330006, China; ^3^Department of Rheumatology, The First Affiliated Hospital of Nanchang University, Nanchang, Jiangxi, China

## Abstract

This study is aimed at exploring the levels of peripheral blood circular RNAs (circRNAs) as biomarker candidates for the diagnosis of new-onset rheumatoid arthritis (RA). The selected twenty-two circRNAs in peripheral blood from new-onset RA patients and healthy controls (HC) were determined by quantitative reverse transcription-polymerase chain reaction (qRT-PCR). The levels of hsa_circ_0002715, hsa_circ_0001947, hsa_circ_0000367, and hsa_circ_0035197 were significantly increased in the peripheral blood of new-onset RA patients than in the peripheral blood of HC. And, there were obvious differences in the above four peripheral blood circRNAs between new-onset RA patients and systemic lupus erythematosus (SLE) patients and ankylosing spondylitis (AS) patients. Moreover, there were obvious differences in hsa_circ_0001947 and hsa_circ_0035197 between new-onset RA patients and patients with undiagnosed arthritis (UA). Receiver operating characteristic (ROC) curve analysis suggested that the levels of hsa_circ_0002715 and hsa_circ_0000367 in peripheral blood could distinguish new-onset RA patients from the HC, AS patients, and SLE patients, and the levels of hsa_circ_0001947 and hsa_circ_0035197 in peripheral blood could distinguish new-onset RA patients from the HC, AS patients, SLE patients, and UA patients. The logistic regression model showed that the combination of hsa_circ_0002715 and hsa_circ_0035197 could provide the best diagnostic accuracy with an area under the curve (AUC) of 0.758 (sensitivity: 72.9%, specificity: 71.4%). Moreover, the levels of peripheral blood hsa_circ_0002715 were correlated with swollen joint count (SJC), tender joint count (TJC), disease duration, rheumatoid factor (RF), anticitrullinated protein antibodies (ACPA), and hematologic disorder. And, the levels of peripheral blood hsa_circ_0035197 were correlated with hematologic disorder. This study suggests that the combination of hsa_circ_0002715 and hsa_circ_0035197 in peripheral blood may be a potential biomarker of patients with new-onset RA and may be associated with disease activity.

## 1. Introduction

Rheumatoid arthritis (RA) is the most common chronic and debilitating systemic autoimmune disease characterized by synovitis, destruction of the joints, and systemic immune and inflammatory manifestations. Although the treatment and survival rate of patients with RA have improved, most patients experience long-term joint damage, severe illness, and disability [1]. Current diagnostic methods, including American College of Rheumatology (ACR) classification criteria [2], anticitrullinated protein antibodies (ACPA), and rheumatoid factor (RF), show various disadvantages for the early diagnosis of RA. This may cause early RA patients to be misdiagnosed, and the untimely treatment may lead to a worse clinical outcome [3]. Therefore, new biomarkers aimed at improving the diagnosis and prognosis evaluation of RA will be highly valuable.

Circular RNAs (circRNAs), a unique form of RNA, possess covalently closed continuous loops without free ends [4, 5]. This confers resistance to RNase R, allowing circRNAs to be selectively enriched during sample processing and making them more suitable biomarkers than other types of RNA [6, 7]. Increasing evidences have revealed that circRNAs can act as microRNA (miRNA) “sponges” to regulate the expression of genes encoding proteins [8–10]. A growing number of studies demonstrated that the dysregulation of circRNAs is involved in the development of various human diseases, such as atherosclerotic vascular diseases, diabetes mellitus, Alzheimer's disease, and cancer [11–13]. Recent studies have also confirmed that circRNAs play a crucial role in the occurrence of autoimmune disease, such as systemic lupus erythematosus (SLE) and primary biliary cholangitis [14, 15]. However, little is known about the roles of circRNAs for the diagnosis and prognosis evaluation of patients with RA. Recently, some studies have demonstrated that peripheral blood mononuclear cell (PBMC) circRNAs are involved in the pathogenesis of RA [16, 17]. For instance, hsa_ circ_104871 in PBMCs has been reported to be a potential biomarker of RA [16]. Besides, our previous researches have revealed that peripheral blood hsa_circ_0044235 could regulate the expression of mir-892a and can serve as a potential diagnostic biomarker of RA [18]. Therefore, further research of circRNAs in RA is warranted.

In one of our previous studies, we found some differentially expressed circRNAs in the peripheral blood from SLE patients by circRNA microarray screening, which suggests that circRNAs might play a role in autoimmune diseases. Moreover, we found some differentially expressed circRNAs between RA patients and healthy controls (HC); we also found that hsa_circ_0044235 can serve as a potential diagnostic biomarker of RA. Therefore, some other dysregulated circRNAs were selected to investigate the possibility of being used as diagnosis biomarkers for distinguishing new-onset RA patients from SLE patients, ankylosing spondylitis (AS) patients, undiagnosed arthritis (UA) patients, and HC in this study. Results showed that the levels of hsa_circ_0002715, hsa_circ_0000367, hsa_circ_0001947, and hsa_circ_0035197 in patients with new-onset RA were significantly increased. And, the levels of hsa_circ_0002715 were correlated with swollen joint counts (SJC), tender joint counts (TJC), and some autoantibodies of new-onset RA patients. Moreover, a logistic regression model showed that a combination of hsa_circ_0002715 and hsa_circ_0035197 could provide the best diagnostic accuracy. In conclusion, hsa_circ_0002715 and hsa_circ_0035197 in peripheral blood were found to have potential to be used as new biomarkers for new-onset RA diagnosis.

## 2. Materials and Methods

### 2.1. Participants

59 new-onset RA patients receiving clinical care at the Department of Rheumatology, the First Affiliated Hospital of Nanchang University from September 2018 to February 2019 were enrolled in this study. All RA patients fulfilled the revised ACR criteria for RA [2], i.e., the patients enrolled were those with new-onset rheumatoid arthritis who did not receive therapy with disease-modifying antirheumatic drugs (DMARDs) before specimens were collected. Among all RA patients, 8 patients were reexamined after one month of immunosuppressive treatment by using glucocorticoids or NSAIDs (nonsteroidal anti-inflammatory drugs). Thirty-five age- and sex-matched HC who were free from autoimmune or inflammatory diseases and who were unrelated to the patients were recruited from the Physical Examination Center of the First Affiliated Hospital of Nanchang University from September 2018 to February 2019. In addition, 25 AS patients fulfilled the modified New York criteria [19], 48 SLE patients fulfilled the revised ACR criteria for SLE [20], and 19 patients with early UA were also enrolled from the First Affiliated Hospital of Nanchang University from September 2018 to February 2019. All study protocols were approved by the Ethics Committee of the First Affiliated Hospital of Nanchang University (no. 019). All participants in this study were informed and signed written consent.

### 2.2. Preparation of Peripheral Blood Samples and RNA Isolation

Peripheral blood samples (2 ml) were collected into EDTA-2K-containing tubes and total RNA was extracted as soon as possible by using the TRIzol Reagent according to the manufacturer's protocol. The concentration and quality of the RNA were assessed by absorbance spectrometry measuring absorbance ratios of A260/A280 and A 260/A230 using a NanoDrop ND-1000 spectrophotometer (Agilent, Santa Clara, CA, USA). Isolated total RNA was kept at -80°C or immediately used for reverse transcription.

### 2.3. Quantitative Reverse Transcription-Polymerase Chain Reaction (qRT-PCR) Analysis

According to our previous study [18], qRT-PCR was performed following the manufacturer's instructions. The primers used in qRT-PCR are shown in Table 1. *β*-Actin was set as an internal control. The relative expression level of each circRNA was measured through the equation 2^−ΔΔCt^. The experiments were repeated at least three times.

### 2.4. Clinical Assessments and Laboratory Index

Data of SJC, TJC, and visual analogue scale (VAS) for all patients were recorded at the time of recruitment and one month after treatment. RA disease activity was measured according to the disease activity score 28 (DAS28) [21]. The DAS28 can be calculated based on SJC, TJC, VAS, and erythrocyte sedimentation rate (ESR) (or alternatively C-reactive protein (CRP)). ESR was determined according to the instructions described by the manufacturer. CRP and RF were measured by nephelometry. ACPA of immunoglobulin (Ig) G class in serum were measured by commercially available enzyme-linked immunosorbent assay (ELISA) kits (Kexin, Shanghai, China). Blood routine parameters including white blood cell count (WBC), red blood cell count (RBC), hemoglobin (HGB), hematocrit (HCT), mean corpuscular volume (MCV), mean corpuscular hemoglobin (MCH), mean corpuscular hemoglobin concentration (MCHC), red blood cell volume distribution width (RDW), platelet count (PLT), mean platelet volume (MPV), plateletcrit (PCT), platelet distributing width (PDW), lymphocyte count (*L*), lymphocyte percentage (*L*%), monocyte count (*M*), monocyte percentage (*M*%), neutrophil count (*N*), neutrophil percentage (*N*%), eosinophil count (*E*), eosinophil percentage (*E*%), basophil count (*B*), basophil percentage (*B*%), and platelet large cell ratio (P-LCR) were measured using the Sysmex XE-2100 analyzer (Sysmex, Kobe, Japan).

### 2.5. Statistical Analysis

Baseline characteristics were assessed using descriptive statistics. Student's *t*-test and Mann-Whitney's *U* test were employed to compare normally distributed parameters and those with skewed distribution, respectively. For the evaluation of changes with treatment, paired *t*-tests or Wilcoxon's matched-pairs test was used. Likewise, the Pearson method or the nonparametric Spearman method was used for correlation analysis. In addition, receiver operating characteristic (ROC) curves were carried out to assess the diagnostic value of dysregulated circRNAs.

Two-sided *P* values < 0.05 were considered statistically significant. Statistical analysis and graphic presentation were carried out with SPSS version 16.0 (SPSS Inc., Chicago, IL, US) and GraphPad Prism version 5.0 (GraphPad Software, San Diego, CA, USA).

## 3. Results

### 3.1. Characteristics of the Study Population

**Figure 1:**
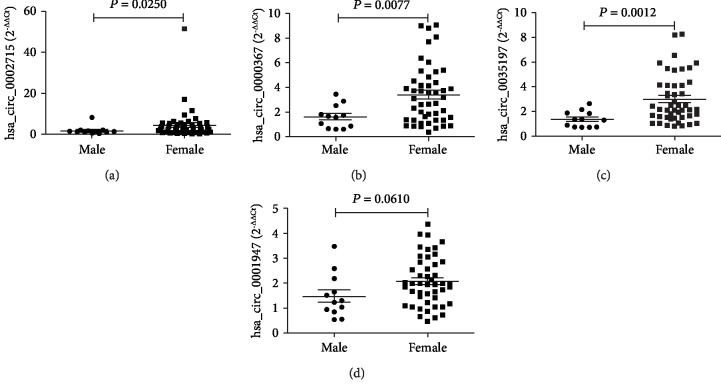
Gender-to-gender comparison of circRNA levels in peripheral blood of new-onset RA patients. The levels of peripheral blood hsa_circ_0002715 (a), hsa_circ_0000367 (b), and hsa_circ_0035197 (c) were significantly higher in new-onset female RA patients than in new-onset male RA patients, but no differences were found in hsa_circ_0001947 levels (d). Rheumatoid arthritis (RA).

A total of 186 participants were enrolled in this study, including 59 patients with new-onset RA, 35 HC, 25 patients with AS, 48 patients with SLE, and 19 patients with UA. All RA patients were new-onset patients with no history of corticosteroids or immunosuppressive drug use before registration. The demographic characteristics of the study population are shown in Table 2. There was no significant difference in the age and gender between the new-onset RA group, the UA group, and the HC group. There was no significant difference in the gender between the new-onset RA group and the SLE group. Patients with new-onset RA, AS, and SLE were not age matched, and patients with new-onset RA and AS were not gender matched in the present study. No correlation between circRNA levels and age was observed in HC and in new-onset RA, AS, SLE, and UA patients (data not shown). No correlation between circRNA levels and gender was observed in HC and in AS, SLE, and UA patients (data not shown). However, the levels of hsa_circ_0002715, hsa_circ_0000367, and hsa_circ_0035197 were significantly higher in new-onset female RA patients than in new-onset male RA patients (all *P* < 0.05) (Figure 1).

### 3.2. Screening of Abnormal Expression circRNAs in Peripheral Blood from New-Onset RA Patients and HC

**Figure 2:**
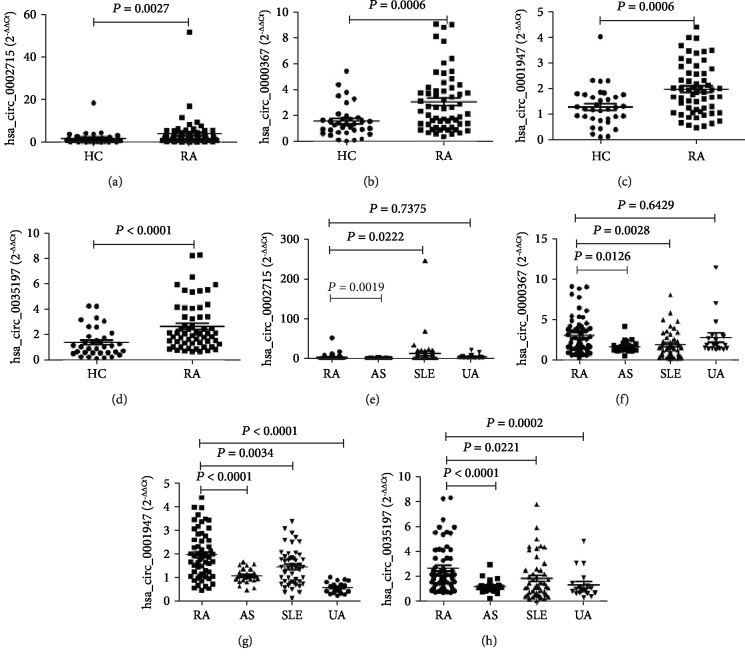
The levels of peripheral blood hsa_circ_0002715, hsa_circ_0000367, hsa_circ_0001947, and hsa_circ_0035197 in new-onset RA patients. The levels of peripheral blood hsa_circ_0002715 (a, e), hsa_circ_0000367 (b, f), hsa_circ_0001947(c, g), and hsa_circ_0035197 (d, h) were significantly higher in new-onset RA patients than in HC and AS patients. The levels of peripheral blood hsa_circ_0002715 (e) were significantly lower in new-onset RA patients than in SLE patients, while the levels of peripheral blood hsa_circ_0000367 (f), hsa_circ_0000367 (g), and hsa_circ_0035197 (h) were significantly higher in new-onset RA patients than in SLE patients. The levels of peripheral blood hsa_circ_0001947 (g) and hsa_circ_0035197 (h) were significantly higher in new-onset RA patients than in UA patients, while the levels of peripheral blood hsa_circ_0002715 (e) and hsa_circ_0000367 (f) showed no significant difference in the peripheral blood of new-onset RA patients and UA. Ankylosing spondylitis (AS), healthy controls (HC), rheumatoid arthritis (RA), systemic lupus erythematosus (SLE), and undiagnosed arthritis (UA).

Twenty-two differentially expressed circRNAs (hsa_circ_0004156, hsa_circ_0027070, hsa_circ_0046995, hsa_circ_0002715, hsa_circ_0008675, hsa_circ_0031482, hsa_circ_0082689, hsa_circ_0082626, hsa_circ_0082688, hsa_circ_0001093, hsa_circ_0022383, hsa_circ_0001226, hsa_circ_0010932, hsa_circ_0002473, hsa_circ_0038651, hsa_circ_0000691, hsa_circ_0001947, hsa_circ_0079787, hsa_circ_0000367, hsa_circ_0068784, hsa_circ_0035197, and hsa_circ_0072568) between SLE and HC in circRNA microarrays were selected to investigate their levels in peripheral blood from RA patients and HC. As shown in Figure 2, the levels of 4 circRNAs (hsa_circ_0002715, hsa_circ_0000367, hsa_circ_0001947, and hsa_circ_0035197) were significantly upregulated in the peripheral blood of new-onset RA patients compared with those in HC (*P* < 0.05), and the levels of other circRNAs showed no significant difference in the peripheral blood of new-onset RA patients and HC (*P* > 0.05) (data not shown). Furthermore, the levels of these 4 circRNAs were also significantly upregulated in the peripheral blood of new-onset RA patients compared with those in AS patients (*P* < 0.05). 3 circRNAs (hsa_circ_0000367, hsa_circ_0001947, and hsa_circ_0035197) in the peripheral blood of new-onset RA patients were significantly upregulated compared with those in SLE patients (*P* < 0.05), but hsa_circ_0002715 was significantly lower than that in SLE patients (*P* < 0.05). In addition, 2 circRNAs (hsa_circ_0001947 and hsa_circ_0035197) in the peripheral blood of new-onset RA patients were significantly upregulated compared with those in UA patients (*P* < 0.05), but the levels of hsa_circ_0002715 and hsa_circ_0000367 showed no significant difference in the peripheral blood of new-onset RA patients and UA (*P* > 0.05).

### 3.3. Potential Diagnostic Values of Hsa_circ_0002715, Hsa_circ_0000367, Hsa_circ_0001947, and Hsa_circ_0035197 in Peripheral Blood in New-Onset RA

**Figure 3:**
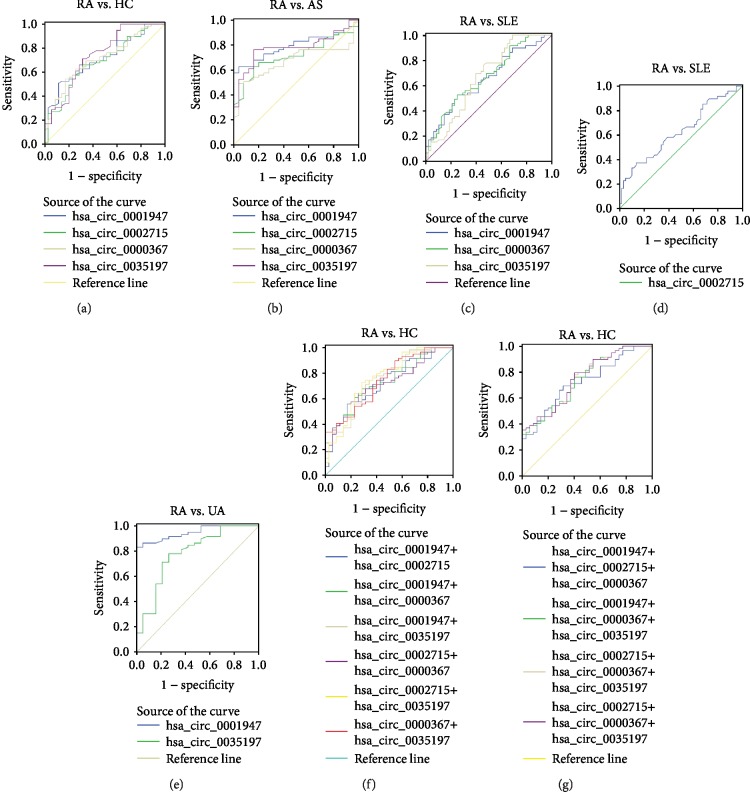
ROC analysis of differentially expressed circRNAs in peripheral blood from new-onset RA patients. Diagnostic power of differentially expressed circRNAs in peripheral blood for RA vs. HC (a), RA vs. AS (b), RA vs. SLE (c, d), and RA vs. UA (e) was determined by ROC curve analysis. The results show the AUC for the sensitivity and specificity of each circRNA. The improved AUC for the combination of circRNAs are shown in (f, g). Area under the curve (AUC), ankylosing spondylitis (AS), circular RNAs (circRNAs), healthy controls (HC), receiver operating characteristic (ROC), and systemic lupus erythematosus (SLE).

The above results showed that the levels of hsa_circ_0002715, hsa_circ_0000367, hsa_circ_0001947, and hsa_circ_0035197 in peripheral blood of new-onset RA patients were significantly different from those of HC, AS patients, SLE patients, and UA patients. Then, we investigated whether circRNAs could be used as a new diagnostic marker of new-onset RA using the ROC curve analysis. As shown in Figure 3 and Table 3, the area under the curve (AUC) values showed that the levels of hsa_circ_0002715 and hsa_circ_0000367 in peripheral blood could differentiate new-onset RA patients from the HC, AS patients, and SLE patients, and the levels of hsa_circ_0001947 and hsa_circ_0035197 in peripheral blood could differentiate new-onset RA patients from the HC, AS patients, SLE patients, and UA patients. The highest AUC was found for hsa_circ_0035197 (AUC: 0.742), followed by hsa_circ_0000367 (AUC: 0.713), hsa_circ_0001947 (AUC: 0.709), and hsa_circ_0002715 (AUC: 0.686) in differentiating new-onset RA from HC. And, the highest AUC was found for hsa_circ_0001947 (AUC: 0.793), followed by hsa_circ_0035197 (AUC: 0.779), hsa_circ_0002715 (AUC: 0.715), and hsa_circ_0000367 (AUC: 0.673) in differentiating new-onset RA from AS. In addition, the highest AUC was found for hsa_circ_0000367 (AUC: 0.669), followed by hsa_circ_0035197 (AUC: 0.667), hsa_circ_0001947 (AUC: 0.654), and hsa_circ_0002715 (AUC: 0.629) in differentiating new-onset RA from SLE. Moreover, the highest AUC was found for hsa_circ_0001947 (AUC: 0.947), followed by hsa_circ_0035197 (AUC: 0.781) in differentiating new-onset RA from UA.

To evaluate the cumulative performances of these four circRNAs in discriminating new-onset RA from HC, a binary logistic regression was performed. As shown in Figure 3 and Table 4, the logistic regression model showed that the combination of hsa_circ_0002715 and hsa_circ_0035197 could provide the best diagnostic accuracy, with the AUC of 0.758 (sensitivity: 72.9%, specificity: 71.4%). The combination of all these four circRNAs, any three circRNAs, and any two circRNAs had no improvement in new-onset RA diagnosis when compared with the aforementioned combination of two circRNAs.

### 3.4. Correlation between Peripheral Blood circRNA Levels in New-Onset RA and Clinical Variables

**Figure 4:**
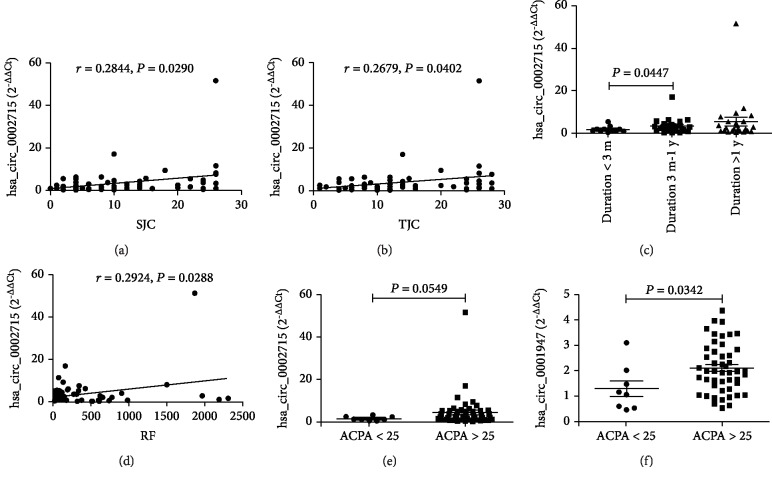
Correlation between the levels of peripheral blood circRNAs in new-onset RA and clinical variables. The levels of peripheral blood hsa_circ_0002715 were correlated with SJC (a), TJC (b), disease duration (c), RF (d), and ACPA (e). The levels of peripheral blood hsa_circ_0001947 were correlated with ACPA (f). Anticitrullinated protein antibodies (ACPA), circular RNAs (circRNAs), rheumatoid factors (RF), swollen joint count (SJC), and tender joint count (TJC).

To determine whether these 4 peripheral blood circRNAs from new-onset RA patients were relevant biomarkers for the activity of RA, the relationship between circRNA levels in peripheral blood and ESR, CRP, DAS28, VAS, SJC, and TJC and duration of new-onset RA patients were explored. Of the 4 circRNAs with an abnormal expression in peripheral blood from RA patients, only one circRNA was found to be associated with some relevant biomarkers for the disease activity of new-onset RA. The levels of peripheral blood hsa_circ_0002715 were correlated with SJC and TJC of new-onset RA patients (*r* = 0.2844, *P* = 0.0290; *r* = 0.2679, *P* = 0.0402) (Figures 4(a) and 4(b)). And, the levels of all these 4 peripheral blood circRNAs tend to elevate in patients with disease duration longer than 3 months, but a significant difference was not reached (data not shown). Moreover, we found that the levels of peripheral blood hsa_circ_0002715 were significantly increased in patients with a disease duration of longer than 3 months and less than 1 year (1 y > disease duration > 3 m) compared to patients with a disease duration of less than 3 months (*P* = 0.0447) (Figure 4(c)). There was no correlation between the levels of other circRNAs in peripheral blood and ESR, CRP, DAS28, and VAS of new-onset RA patients. In addition, we performed a one-month follow-up evaluation in 8 RA patients who received regular treatment with immunosuppressive drugs and corticosteroids or NSAIDs. The clinical response and levels of these peripheral blood circRNAs were monitored during the course of treatment, but no difference was found (data not shown).

RA is characterized by the overproduction of such autoantibodies as RF and ACPA. Thus, the hallmark antibodies of RA including RF and ACPA were determined and analyzed for their correlation with the levels of these 4 peripheral blood circRNAs from new-onset RA patients. Data showed that the levels of peripheral blood hsa_circ_0002715 were correlated with RF (*r* = 0.2924, *P* = 0.0288) (Figure 4(d)). And, the levels of peripheral blood hsa_circ_0002715 were slightly increased in patients with positive ACPA (*P* = 0.0549) (Figure 4(e)). Moreover, the levels of peripheral blood hsa_circ_0001947 were significantly increased in patients with positive ACPA (*P* = 0.0342) (Figure 4(f)). There was no correlation between the levels of other circRNAs in peripheral blood and RF and ACPA.

As shown in Table 5, the levels of peripheral blood hsa_circ_0002715 in new-onset RA patients were associated with WBC, RBC, HGB, HCT, and L. The levels of peripheral blood hsa_circ_0000367 in new-onset RA patients were associated with M, M%, and PLT. The levels of peripheral blood hsa_circ_0001947 in new-onset RA patients were associated with L%, M%, and N%. The levels of peripheral blood hsa_circ_0035197 in new-onset RA patients were associated with M and M%. Additionally, we found that the levels of peripheral blood hsa_circ_0000367 in HC were associated with RBC, HGB, and HCT.

### 3.5. Target miRNA Analysis of Hsa_circ_0002715, Hsa_circ_0000367, Hsa_circ_0001947, and Hsa_circ_0035197

To determine the function of candidate biomarker circRNAs, we predicted the target miRNAs by aligning with the MREs of differentially expressed circRNAs using miRanda software. As shown in Table 6, five putative miRNA targets of hsa_circ_0002715, hsa_circ_0001947, hsa_circ_0000367, and hsa_circ_0035197 were found, respectively.

## 4. Discussion

CircRNAs are ubiquitous and functionally important. As the expression and function of circRNAs during the development of RA are still largely elusive, we examined 22 differentially expressed circRNAs in the peripheral blood to detect circRNAs associated with new-onset RA using qRT-PCR. Four circRNAs (hsa_circ_0002715, hsa_circ_0000367, hsa_circ_0001947, and hsa_circ_0035197) were chosen for further analysis because of their differential expression between new-onset RA patients and HC.

Hsa_circ_0002715, hsa_circ_0000367, hsa_circ_0001947, and hsa_circ_0035197 in the peripheral blood of new-onset RA patients were significantly increased compared to those in the peripheral blood of HC. In addition, hsa_circ_0002715 and hsa_circ_0000367 were abnormally expressed between new-onset RA, SLE, and AS. Hsa_circ_0001947 and hsa_circ_0035197 were abnormally expressed between new-onset RA and other autoimmune diseases, such as SLE, UA, and AS. These results indicated that hsa_circ_0002715, hsa_circ_0000367, hsa_circ_0001947, and hsa_circ_0035197 are not only associated with RA but they also have potential to effectively distinguish RA patients from AS patients and SLE patients.

circRNAs are very stable in blood circulation. Moreover, the expression of circRNAs has good tissue and developmental stage specificity. These characteristics make circRNAs very suitable to be used as a new serum marker for a variety of diseases [22]. The stability can be explained by the special characteristic of circRNAs, which is a type of closed circular RNA and is free of exonuclease-mediated degradation [23]. Here, we used an ROC curve to evaluate the clinical diagnostic value in the diagnosis of new-onset RA of these four circRNAs and found that the AUC of hsa_circ_0035197 (AUC: 0.742) is the highest, followed by hsa_circ_0000367, hsa_circ_0001947, and hsa_circ_0002715. Moreover, the logistic regression model showed that the combination of hsa_circ_0002715 and hsa_circ_0035197 could provide the best diagnostic accuracy. These results indicated that these four circRNAs, especially the combination of hsa_circ_0002715 and hsa_circ_0035197 in peripheral blood have potential to be diagnostic biomarkers for new-onset RA.

Our results also showed that the levels of peripheral blood hsa_circ_0002715 are correlated with SJC, TJC, disease duration, RF, WBC, RBC, HGB, HCT, and L of new-onset RA patients; the levels of peripheral blood hsa_circ_0001947 are correlated with ACPA, L%, M%, and N% of new-onset RA patients; the levels of peripheral blood hsa_circ_0000367 are correlated with PLT, M, and M% of new-onset RA patients; and the levels of peripheral blood hsa_circ_0035197 are correlated with M and M% of new-onset RA patients. These results showed that the levels of peripheral blood hsa_circ_0002715, hsa_circ_0001947, hsa_circ_0000367, and hsa_circ_0035197 are associated with SJC, TJC, disease duration, autoantibodies, and hematological system damage of RA, indicating that these circRNAs may be relevant biomarkers for disease activity. Other reports [16] and our previous report [18] showed that the levels of circRNAs were not correlated with biomarkers for disease activity of RA, such as DAS28, CRP, ESR, RF, or ACPA [24–26]. The discrepancy of these results can be partially explained by the heterogeneity of disease, differences in disease duration, and ongoing treatments.

By searching circBase, a database for circRNAs, we found that hsa_circ_0002715, hsa_circ_0001947, hsa_circ_0000367, and hsa_circ_0035197 regulate the expression of the pericentrin (PCNT) gene, the AF4/FMR2 family member 2 (AFF2) gene, the sialic acid acetylesterase (SIAE) gene, and the ATPase phospholipid transporting 8B4 (ATP8B4) gene, respectively. PCNT is an integral component of the pericentriolar material, which binds to calmodulin and is expressed in the centrosome. It was reported that PCNT is associated with hematological abnormalities [26]. Our results and other reports [27] have indicated that the hematological system of RA was impaired. Considering these, we speculated that PCNT may play a role in RA. The AFF2-encoding gene is associated with the folate-sensitive fragile X E locus on chromosome X, which was reported to cause fragile X E syndrome. However, to our knowledge, no study on the role of AFF2 in RA has been published until now. SIAE is an enzyme which catalyzes the removal of 9-O-acetylation modifications from sialic acids. Previous studies have demonstrated that some mutations in this gene are associated with susceptibility to common autoimmune disease, such as juvenile idiopathic arthritis [28]. However, Zhang et al. [29] found no association between SIAE gene mutation and RA in a Han Chinese population. Therefore, the detailed role of SIAE in RA needs further investigation. ATP8B4 is a member of the cation transport ATPase (P-type) family, type IV subfamily, which is speculated to participate in ATP biosynthesis and phospholipid transport via a variety of unknown mechanisms. *ATP8B4* was recently identified as a risk factor for systemic sclerosis, which is a rare multisystem autoimmune disease [30]. However, no report about the role of *ATP8B4* in RA has been published until now. The interactions between hsa_circ_0002715, hsa_circ_0001947, hsa_circ_0000367, and hsa_circ_0035197 and the above potential target genes remain largely unknown and require further research.

It was reported that the levels and functions of circRNAs are closely related to the occurrence and development of autoimmune disease, such as RA [18] and SLE [14]. It has been proven that circRNAs function by regulating the levels of target miRNAs through a molecular sponge mechanism and then regulating the expression of corresponding target genes of miRNAs. To the best of our knowledge, no study has definitively demonstrated the biofunction of these verified circRNAs in RA patients. It has been shown that the MREs of circRNAs can bind matched miRNA and thereby reduce miRNA-mediated posttranscriptional repression. To investigate the function of hsa_circ_0002715, hsa_circ_0001947, hsa_circ_0000367, and hsa_circ_0035197, the potential miRNA targets of these circRNAs were predicted by using Arraystar's miRNA target prediction software. Five putative miRNAs targets of hsa_circ_0002715, hsa_circ_0001947, hsa_circ_0000367, and hsa_circ_0035197 were found. To the best of our knowledge, hsa-miR-378d and hsa-miR-26b-3p have been shown to correlate with rheumatoid arthritis development [31, 32]. However, no report about the role of the other putative miRNA targets in RA has been published. Therefore, further research is needed to confirm the role of the above putative miRNA targets in RA and whether circRNAs (hsa_circ_0002715, hsa_circ_0001947, hsa_circ_0000367, and hsa_circ_0035197) play a role in RA by interacting with the above putative miRNA targets.

In this study, we aimed to show the possible association of peripheral blood circRNAs with new-onset RA. Our results demonstrated that peripheral blood hsa_circ_0002715, hsa_circ_0001947, hsa_circ_0000367, and hsa_circ_0035197 were differentially expressed between new-onset RA patients, AS patients, SLE patients, and HC. In addition, we found that the combination of hsa_circ_0002715 and hsa_circ_0035197 in the peripheral blood may be a potential biomarker for patients with new-onset RA and the levels of these circRNAs associate with the disease activity of RA.

## Figures and Tables

**Table 1:** Specific circRNA primers used for qRT-PCR analysis.

circRNAs	Primer sequence (F)	Primer sequence (R)
Hsa_circ_0004156	TGCAGCATCCCAGTTTTTGG	GCCTGCTTCATCTTTATGCACT
Hsa_circ_0027070	GCACAAGAGCCTTGATTGAAGA	TGGCCAGGGTAGGCTGATA
Hsa_circ_0046995	TCAGTTCCATCCGGGTCATC	AGCTTGGAAATGATTCTTCTGCT
Hsa_circ_0002715	GCAAACCTCCTCTCCATGCT	GTGAAAAGGCTGTGCCTGTG
Hsa_circ_0008675	GGAAGCCTTGCAGTTTGCTC	AGCATTGGCTGGTGGGTTAT
Hsa_circ_0031482	ACACTTTAACCACCAGCCTCA	TCCAAAAGCAGAGGCCCAGT
Hsa_circ_0082689	GTCCCCAAACACTCTTAGCCA	CACACTCAGGTTGTGTTCGG
Hsa_circ_0082626	TCACCAAGCCAGCCAATTCT	TCAGTCCAGAGAGTTCGTGA
Hsa_circ_0082688	TGCCGTATCGATGGCAATTC	ATAGCTCAGGTGGTCAACGC
Hsa_circ_0001093	CTGACACCCAAGCAGTCAAT	TCTCCAGGGAAGAGTCCCAAA
Hsa_circ_0022383	CCACAAGGATCCCGATGTGAA	TTCACCAATCAGCAGGGGTT
Hsa_circ_0001226	CGATCTGCTTCTCCAGGTTGT	AGGTGGCCGACATGAAAAAGA
Hsa_circ_0010932	ACCCTGGAATTGCTGTATTTTTCC	GAGCACCTGTTTGACCCAGA
Hsa_circ_0002473	TGGACTTCACTGCAGCAAGATT	GTGCAGCTTTTGATTTGCCC
Hsa_circ_0038651	CCGCGGCCGCATCTAC	TGGTCCGTGCCACACAG
Hsa_circ_0000691	AGACCATTCCATTCTGGCTACAG	TGTTCAGGGGAAGGTCACTG
Hsa_circ_0001947	ACACTCTTGGATGGAAAACCCA	CGTGTTCTGGACTCGGTTGG
Hsa_circ_0079787	AGAGGAAGTTTGATTGCACTCTG	TTCACGGAGAGGTTGTGTCC
Hsa_circ_0000367	AGACTGGCGTGAAACCTTCC	TGCACAGTGGATGGATCATGG
Hsa_circ_0068784	GGGTGTTGCTATCTCTAACCCA	TGGCCACATCCCTGAATGTTA
Hsa_circ_0035197	TAATCGGCAGTCTGAAGTGCT	GGCATTTGCCACTCTTTGGA
Hsa_circ_0072568	GCAAGATCGAGCACCTAGCA	GCTTGGAGAATTAGCCCGGA
*β*-Actin	CATGTACGTTGCTATCCAGGC	CTCCTTAATGTCACGCACGAT

Circular RNAs (circRNAs) and quantitative reverse transcription-polymerase chain reaction (qRT-PCR).

**Table 2:** Clinical details of the patients with new-onset RA, AS, SLE, and HC.

Clinical characteristics	RA	HC	AS	SLE	UA
Number of subjects	59	35	25	48	19
Sex (male/female)	12/47	12/23	17/8	4/44	6/13
Age (years)	48.61 ± 13.30	46.29 ± 14.16	33.04 ± 8.96	43.12 ± 17.12	49.05 ± 11.82
Duration (day)	1479.27 ± 2314.91				
DAS28	6.48 ± 1.51				
SJC	12.22 ± 8.40				
TJC	13.81 ± 8.40				
VAS	68.95 ± 12.02				
ESR (mm/h)	53.72 ± 34.90				
CRP (mg/l)	30.63 ± 35.19				
RF (IU/ml)	470.48 ± 573.48				
ACPA (RU/ml)	875.73 ± 909.06				
WBC (10^9^/l)	8.08 ± 2.58^∗^	5.81 ± 0.81	7.43 ± 1.68	6.33 ± 3.19^#^	6.34 ± 2.10^^^
RBC (10^12^/l)	4.41 ± 0.53	4.54 ± 0.41	4.72 ± 0.74^&^	3.64 ± 0.90^#^	4.59 ± 0.55
HGB (g/l)	123.37 ± 20.86^∗^	138.94 ± 12.32	140.20 ± 20.08^&^	119.33 ± 83.69^#^	132.58 ± 14.63
HCT (l/l)	0.38 ± 0.05^∗^	0.41 ± 0.03	0.42 ± 0.06^&^	0.32 ± 0.08^#^	0.41 ± 0.04^^^
PLT (10^9^/l)	328.85 ± 129.63^∗^	246.74 ± 41.91	297.60 ± 59.87	194.77 ± 82.42^#^	229.89 ± 61.65^^^
*L* (10^9^/l)	1.60 ± 0.57^∗^	1.90 ± 0.33	2.08 ± 0.61^&^	1.31 ± 0.77^#^	1.82 ± 0.55
*L*%	21.34 ± 8.54^∗^	33.02 ± 5.28	26.74 ± 8.09^&^	22.97 ± 11.03	30.04 ± 8.07^^^
*M* (10^9^/l)	0.43 ± 0.17^∗^	0.34 ± 0.06	0.41 ± 0.15	0.49 ± 0.37	0.37 ± 0.13
*M*%	5.58 ± 2.20	5.82 ± 1.08	5.52 ± 1.71	8.09 ± 4.27^#^	5.89 ± 1.22
*N* (10^9^/l)	5.91 ± 2.45^∗^	3.43 ± 0.68	4.76 ± 1.30^&^	4.48 ± 2.83^#^	4.04 ± 1.67^^^
*N*%	71.21 ± 10.90^∗^	58.83 ± 5.78	63.86 ± 7.42^&^	68.18 ± 13.21	62.24 ± 8.62^^^

^∗^ *P* < 0.05: RA compared to HC; ^&^*P* < 0.05: AS compared to RA; ^#^*P* < 0.05: SLE compared to RA; ^^^*P* < 0.05: UA compared to RA. Anticitrullinated protein antibodies (ACPA); ankylosing spondylitis (AS); C-reactive protein (CRP); disease activity score (DAS28); erythrocyte sedimentation rate (ESR); healthy controls (HC); hematocrit (HCT); hemoglobin (HGB); lymphocyte count (*L*); lymphocyte percentage (*L*%); monocyte count (*M*); monocyte percentage (*M*%); neutrophil count (*N*); neutrophil percentage (*N*%); platelet count (PLT); rheumatoid arthritis (RA); red blood cell count (RBC); rheumatoid factors (RF); systemic lupus erythematosus (SLE); swollen joint count (SJC); tender joint count (TJC); undiagnosed arthritis (UA); visual analogue scale (VAS); white blood cell count (WBC).

**Table 3:** AUC for 4 circRNAs in discriminating new-onset RA patients from HC, AS patients, SLE patients, and UA patients.

Variables	AUC	*P* value	95% C.I.	Sensitivity	Specificity	Cut-off
RA vs. HC						
Hsa_circ_0035197	0.742	<0.0001	0.637-0.848	71.2%	68.6%	>1.454
Hsa_circ_0000367	0.713	0.0006	0.608-0.818	54.2%	82.9%	>2.121
Hsa_circ_0001947	0.709	0.0007	0.604-0.815	50.9%	88.6%	>1.847
Hsa_circ_0002715	0.686	0.0027	0.676-0.796	57.6%	77.1%	>1.847

RA vs. AS						
Hsa_circ_0001947	0.793	<0.0001	0.700-0.886	62.71%	96.0%	>1.578
Hsa_circ_0035197	0.779	<0.0001	0.679-0.878	76.3%	84.0%	>1.315
Hsa_circ_0002715	0.715	0.0019	0.607-0.823	62.7%	84.0%	>1.717
Hsa_circ_0000367	0.673	0.0125	0.560-0.786	49.2%	96.0%	>2.594

RA vs. SLE						
Hsa_circ_0000367	0.669	0.0028	0.566-0.771	52.5%	75.0%	>2.305
Hsa_circ_0035197	0.667	0.0031	0.561-0.773	100.0%	31.3%	>0.678
Hsa_circ_0001947	0.654	0.0063	0.551-0.757	52.5%	75.0%	>1.831
Hsa_circ_0002715	0.629	0.0219	0.522-0.736	86.4%	37.5%	<5.799

RA vs. UA						
Hsa_circ_0001947	0.947	<0.001	0.901-0.992	83.1%	100.0%	>1.014
Hsa_circ_0035197	0.781	0.0002	0.654-0.909	78.0%	73.7%	>1.244
Hsa_circ_0002715	—	—	—	—	—	—
Hsa_circ_0000367	—	—	—	—	—	—

Ankylosing spondylitis (AS); area under the curve (AUC); circular RNAs (circRNAs); healthy controls (HC); rheumatoid arthritis (RA); receiver operating characteristic (ROC); systemic lupus erythematosus (SLE); undiagnosed arthritis (UA).

**Table 4:** Binary logistic regression for 4 circRNAs in discriminating new-onset RA patients from HC.

Variables	AUC	*P* value	95% C.I.	Sensitivity	Specificity
Hsa_circ_0001947+hsa_circ_0002715	0.723	<0.0001	0.618-0.827	55.93	82.86
Hsa_circ_0001947+hsa_circ_0000367	0.727	<0.0001	0.626-0.829	67.8	71.43
Hsa_circ_0001947+hsa_circ_0035197	0.743	<0.0001	0.639-0.847	74.6	65.71
Hsa_circ_0002715+hsa_circ_0000367	0.715	0.0010	0.611-0.819	64.41	74.29
Hsa_circ_0002715+hsa_circ_0035197	0.758	<0.0001	0.656-0.861	72.88	71.43
Hsa_circ_0000367+hsa_circ_0035197	0.745	<0.0001	0.644-0.845	89.83	45.71
Hsa_circ_0001947+hsa_circ_0002715+hsa_circ_0000367	0.727	<0.0001	0.626-0.828	69.49	68.57
Hsa_circ_0001947+hsa_circ_0002715+hsa_circ_0035197	0.754	<0.0001	0.651-0.856	67.8	74.29
Hsa_circ_0002715+hsa_circ_0000367+hsa_circ_0035197	0.754	<0.0001	0.655-0.852	77.97	60
Hsa_circ_0001947+hsa_circ_0000367+hsa_circ_0035197	0.744	<0.0001	0.644-0.845	76.27	60
Hsa_circ_0001947+hsa_circ_0002715+hsa_circ_0000367+hsa_circ_0035197	0.753	<0.0001	0.654-0.851	79.66	60

Area under the curve (AUC); circular RNAs (circRNAs); healthy controls (HC); rheumatoid arthritis (RA).

**Table 5:** Correlation of the expression of 4 circRNAs with other clinical variables of RA and HC.

circRNAs	Variable	*P* value	*r* value
Hsa_circ_0002715 in RA patients	WBC	0.0288	-0.2965
	RBC	0.0226	-0.4058
	HGB	0.0021	-0.3927
	HCT	0.0006	-0.4314
	*L*	0.0355	-0.2744

Hsa_circ_0000367 in RA patients	PLT	0.0113	0.3274
	*M*	0.0072	-0.3438
	*M*%	0.0342	-0.2762

Hsa_circ_0001947 in RA patients	*L*%	0.0126	-0.3229
	*M*%	0.0024	-0.3879
	*N*%	0.0118	0.3257

Hsa_circ_0035197 in RA patients	*M*	0.0027	-0.3834
	*M*%	0.0024	-0.3972

Hsa_circ_0000367 in HC	RBC	0.0496	-0.3344
	HGB	0.0217	-0.3869
	HCT	0.0188	-0.3952

Circular RNAs (circRNAs); healthy controls (HC); hematocrit (HCT); hemoglobin (HGB); lymphocyte count (*L*); lymphocyte percentage (*L*%); monocyte count (*M*); monocyte percentage (*M*%); neutrophil count (*N*); neutrophil percentage (*N*%); platelet count (PLT); rheumatoid arthritis (RA); red blood cell count (RBC); white blood cell count (WBC).

**Table 6:** Putative miRNA targets of hsa_circ_0002715, hsa_circ_0001947, hsa_circ_0000367, and hsa_circ_0035197.

circRNAs	Putative miRNA targets				
Hsa_circ_0002715	Hsa-mir-130b-5p	Hsa-mir-671-3p	Hsa-mir-378a-3p	Hsa-mir-378d	Hsa-mir-127-5p
Hsa_circ_0001947	Hsa-mir-10b-3p	Hsa-mir-26b-3p	Hsa-mir-92a-2-5p	Hsa-mir-488-3p	Hsa-mir-329-5p
Hsa_circ_0000367	Hsa-mir-331-3p	Hsa-mir-4646-5p	Hsa-mir-4797-5p	Hsa-mir-3919	Hsa-mir-3190-3p
Hsa_circ_0035197	Hsa-mir-605-3p	Hsa-mir-101-5p	Hsa-mir-31-5p	Hsa-mir-215-3p	Hsa-mir-766-5p

Circular RNAs (circRNAs); microRNAs (miRNAs).

## Data Availability

The data used to support the findings of this study are available from the corresponding authors upon request.
